# Mebendazole augments sensitivity to sorafenib by targeting MAPK and BCL-2 signalling in n-nitrosodiethylamine-induced murine hepatocellular carcinoma

**DOI:** 10.1038/s41598-019-55666-x

**Published:** 2019-12-13

**Authors:** Nancy S. Younis, Amal M. H. Ghanim, Sameh Saber

**Affiliations:** 1 Department of Pharmaceutical Sciences, College of Clinical Pharmacy, King Faisal University, Al-Ahsa, Kingdom of Saudi Arabia; 2 Department of Pharmacology, Zagazig University, Zagazig, Egypt; 3 Department of Biochemistry, Faculty of Pharmacy, Delta University for Science and Technology, Gamasa, Egypt; 4 Department of Pharmacology, Faculty of Pharmacy, Delta University for Science and Technology, Gamasa, Egypt

**Keywords:** Cancer therapeutic resistance, Receptor pharmacology

## Abstract

Sorafenib (SO) is a multi-kinase inhibitor that targets upstream signals in the MAPK pathway. Drug resistance and transient survival benefits are the main obstacles associated with SO treatment in Hepatocellular carcinoma (HCC) patients. Mebendazole (MBZ), an anthelmintic agent, has demonstrated activity against various cancer types. Therefore, we aimed to investigate the possible mechanisms of MBZ other than its anti-tubulin activity. MBZ (100 mg/kg/day, P.O.) was administered to N-nitrosodiethylamine-induced HCC mice as a monotherapeutic agent or in combination with SO. Our results revealed that MBZ decreased AFP levels, improved liver function and histology and increased survival in HCC mice, particularly when administered in combination with SO. MBZ also reduced hepatic inflammation and fibrogenesis as evidenced by reductions in TNF-α and TGF-β1 levels, respectively. Increased hepatic caspases-3 and -9 and decreased BCL-2 levels suggest induced-cell death. In addition, MBZ demonstrated anti-angiogenic, anti-metastatic, and anti-proliferative effects, as indicated by reduced VEGF levels, MMP-2:TIMP-1 ratios, and reduced cyclin D1 levels and Ki67 immunostaining, respectively. Our main finding was that MBZ targeted downstream signal of the MAPK pathway by inhibiting ERK1/2 phosphorylation. Targeting downstream MAPK signalling by MBZ and upstream signalling by SO is a novel approach to minimizing resistance and prolonging survival.

## Introduction

Hepatocellular carcinoma (HCC) is one of the most serious global health problems. It is the sixth most prevailing neoplasm and is ranked as the third leading cause of cancer-related death^1^. No therapy is sufficiently effective, as most cases are diagnosed at an advanced stage. Moreover, recurrent relapse and drug resistance are serious obstacles in HCC treatment^2,3^. Sorafenib (SO) has been developed as an oral multi-kinase inhibitor that inhibits c-RAF-1 kinase and targets other kinases, such as VEGFR1, VEGFR2, VEGFR3, and PDGFR-β^4^. SO has been clinically approved as a first-line treatment for advanced HCC^5^. However, SO provides transient and modest survival benefits (increasing survival time by approximately 3 months compared to placebo) and high treatment costs^6,7^. Furthermore, HCC becomes resistant to SO within 6 months^8,9^. Therefore, development of effective new strategies for HCC treatment is urgently needed.

The mitogen-activated protein kinase (MAPK) pathway, often recognized as the RAS/RAF/MEK/ERK signalling cascade, is activated in a variety of human cancers, including advanced HCC^10^. This pathway conducts upstream signals to its downstream effectors to regulate physiological processes such as angiogenesis, migration, cell proliferation, differentiation, survival, and death^11,12^. Activation of the MAPK pathway results from binding of growth factors to the tyrosine kinase receptor (TKR), causing a conformational change in the small GTPase RAS, which in turn recruits and activates the serine/threonine kinase RAF. Activated RAF phosphorylates and activates mitogen/extracellular signal-regulated kinase (MEK), whose activation directly leads to the phosphorylation of extracellular signal-regulated kinase (ERK), which is the key effector kinase of this signalling pathway^13^. Phosphorylated ERK1/2 proteins (p-ERK1/2) phosphorylate different transcription factors, which then regulate the expression of proteins involved in angiogenesis, proliferation, extracellular matrix (ECM) production and apoptosis resistance^14,15^.

Accumulating evidence indicates that the RAF/MEK/ERK cascade also has diverse effects on key molecules involved in apoptosis signalling, such as the anti-apoptotic regulatory molecule B-cell lymphoma 2 (BCL-2) and apoptotic regulatory molecules including caspase-3 and caspase-9^16,17^ and affects the expression of many proteins involved in cell cycle regulation (e.g., upregulating cyclin D1, which is excessively expressed in HCC)^18^. This activity in turn leads to cell cycle progression and regulation of apoptosis/survival. Moreover, activated RAS/RAF/MEK/ERK is a common downstream pathway for several growth factors in HCC, such as vascular endothelial growth factor (VEGF), platelet-derived growth factor β (PDGFβ) and transforming growth factor-β (TGF-β)^19,20^. Additionally, persistent activation of ERK in malignant cells can cause enhanced transcription of matrix metalloproteinases (MMPs), which promote invasion and metastasis of HCC cells^21^.

All of the above facts make the MAPK signalling pathway an attractive target for therapeutic intervention in cancer treatment. Selective ERK1/2 inhibitors may have added benefits over MEK inhibitors because ERK is the only downstream target of MEK^22^ and because ERK is the only activator that can stimulate a wide variety of downstream substrates. ERK1/2 inhibitors can oppose the abnormal activation of MAPK signalling induced by upstream RAS mutations^23^. Moreover, it is believed that in the MAPK pathway, ERK inhibitors may be less sensitive to resistance mechanisms than inhibitors of upstream molecules, such as SO. Thus, ERK targeting is considered to be more effective than targeting of other molecules^24^.

Mebendazole (MBZ) has been used safely in humans to treat roundworm and common hookworm infestations. Several studies have suggested that MBZ exhibits anticancer activity against different cancer types^25–27^. However, the mechanisms underlying the antitumour effects of MBZ on HCC have not yet been fully elucidated. Therefore, we aimed to investigate possible mechanisms of MBZ other than its well-recognized anti-tubulin activity when used as a monotherapeutic agent or in combination with SO as a treatment for N-nitrosodiethylamine (DEN)-induced HCC.

## Methods

### Evaluation of *in vitro* cytotoxic activity of MBZ on HepG2 cells

HepG2 cells (American Type Culture Collection (ATCC), HB-8065) were grown in pre-warmed (37 °C) Dulbecco’s modified Eagle’s medium (DMEM) supplemented with 10% foetal bovine serum (FBS), 2 mM glutamine, 100 IU/ml penicillin and 100 µg/ml streptomycin. Then, the cells were sub-cultured and seeded in 96-well plates at a density of 2 × 10^4^ cells/well in 100 µl of DMEM in a humidified atmosphere with 5% CO2 in an incubator. After 24 h, MBZ at a range of concentrations (pre-dissolved in DMSO) was added to each well, and the cells were incubated for 48 h. Cytotoxicity was assayed by adding fifty µl of 3-(4,5 -dimethyl thiazol-2-yl)-2,5-di-phenyl tetrazolium bromide (MTT) in DMEM into each well and incubating the cells for 2.5 h as described by Saber, *et al*.^28^. Then, the medium was removed, fifty µl of propanol was supplemented, and the cells were incubated for 30 minutes with continuous shaking. Absorbance was measured with a microplate reader at 540 nm. The percent growth suppression was calculated as: % growth inhibition = 100 × (mean optical density of untreated HepG2 cells (containing 1% DMSO) - mean optical density of HepG2 cells treated with tested MBZ conc)/mean optical density of untreated HepG2 cells (containing 1% DMSO)). This equation was utilized to assess the cytotoxic properties of MBZ by estimating the half maximal cytotoxic concentration (CTC50; µmol/L).

### Western blot analysis of p-MEK1/2, total MEK1/2 (t-MEK1/2), p-ERK1/2 and total ERK1/2 (t-ERK1/2)

HepG2 cells were sub-cultured and seeded in 6-well culture plates at a density of 1.5 × 10^6^ cells/well. All cells were harvested for western blot analysis of p-MEK1/2, t-MEK1/2, p-ERK, t-ERK, and GAPDH as described by Fabregat, *et al*.^29^. The differential effects of MBZ (0, 0.5, 1 and 5 µmol/L) on the basal levels of p-MEK1/2, MEK1/2, p-ERK1/2 and t-ERK1/2 were determined. After harvesting, cell pellets were lysed in RIPA lysis buffer (Bio Basic, Inc., Markham, Ontario, Canada). The protein conc were determined as previously described by Bradford^30^. The samples (100 µg of protein per lane) were separated by 10% SDS-PAGE (Bio-Rad Laboratories, Inc., USA) and transferred electrophoretically to polyvinylidene difluoride (PVDF) membranes (Bio-Rad Laboratories, Inc.). The membranes were then blocked with a solution of 5% non-fat dried milk in 10 mM Tris -Cl, 100 mM NaCl, and 0.1% Tween 20 (pH 7.5) and incubated overnight at 4 °C with monoclonal antibodies for p-MEK1/2, t-MEK1/2, p-ERK1/2, t-ERK1/2, and GAPDH. The secondary antibody was a rabbit anti-mouse antibody conjugated to horseradish peroxidase (Thermo Fisher Scientific, Inc., USA). Chemiluminescence detection was performed with a chemiluminescent substrate (Clarity^TM^ Western ECL Substrate, Bio-Rad, USA). The chemiluminescent signals were captured using a charge-coupled device (CCD) camera-based ChemiDoc MP imager followed by image analysis to determine the band intensity of the target proteins compared to that for a control sample (0 µmol/L MBZ) after normalization by GAPDH.

### Evaluation of *in vivo* anti-tumour activity

#### Animals

Adult male Swiss albino mice of the CD-1 strain weighing 20 ± 2 g were purchased from the Faculty of Pharmacy, Delta University, Gamasa, Egypt. The animals were housed in a temperature- and humidity-controlled environment under a 12 h:12 h light: dark cycle and given rodent food (4% fat, 23% protein) and water ad lib. All animal care and experimental procedures were approved by the Institutional Animal Care and Use Committee (IACUC) at the Delta University for Science and Technology (Approval Number: FPDU93/2018). All experiments were carried out in accordance with relevant guidelines and regulations.

#### Experimental design

As shown in Table 1, the mice were divided into six groups. The mice in the normal group were administered intraperitoneal (I.P.) injections of saline solution once a week (n = 20) for 120 days. The mice in the normal + MBZ group were administered I.P. injections of saline solution once a week for 120 days and treated with MBZ (100 mg/kg/day P.O.) (Arab Drug Company for Pharmaceutical and Chemical Industries, ADCO, Cairo, Egypt) starting 45 days after the first saline injection until the day 120. The mice in the DEN group were administered I.P. injections of DEN (50 mg/kg) (Sigma Aldrich, St. Louis, MO, USA) once a week (n = 35) for 120 days as described by Saber, *et al*.^31^ with slight modifications. The mice in the SO group were administered SO (30 mg/kg/day P.O.) (Bayer AG, Berlin, Germany) starting 45 days after HCC induction (n = 21) until the day 120. The mice in the MBZ group were administered MBZ (100 mg/kg/day P.O.) starting 45 days after HCC induction (n = 21) until the day 120. Finally, the mice in the MBZ + SO group were administered MBZ (100 mg/kg/day P.O.) plus SO (30 mg/kg/day P.O.) (n = 29) until the day 120.

**Table 1:** Experimental design.

Exp. groups	Days (1:45)	Days (46:120)
Normal group (n = 20)	I.P. injection of saline solution once a week	
Normal + MBZ (n = 20)	I.P. injection of saline solution once a week	I.P. injection of saline solution once a week + MBZ (100 mg/kg/day P.O.)
DEN group (n = 35)	I.P. injection of DEN (50 mg/kg) once a week	I.P. injection of DEN (50 mg/kg) once a week
SO group (n = 21)	I.P. injection of DEN (50 mg/kg) once a week	I.P. injection of DEN (50 mg/kg) once a week + SO (30 mg/kg/day P.O.)
MBZ group (n = 21)	I.P. injection of DEN (50 mg/kg) once a week	I.P. injection of DEN (50 mg/kg) once a week + MBZ (100 mg/kg/day P.O.)
MBZ + SO group (n = 29)	I.P. injection of DEN (50 mg/kg) once a week	I.P. injection of DEN (50 mg/kg) once a week + MBZ (100 mg/kg/day P.O.) + SO (30 mg/kg/day P.O.)

DEN, N-Nitrosodiethylamine; I.P., intraperitoneal; MBZ, mebendazole; p.o., per oral; SO, sorafenib.

#### Rationale of drug dosing

Genetically, the DEN model is a good representation of HCC with poor prognosis^32^. A 1% DEN solution was prepared in normal saline, and MBZ was suspended in distilled water just before use. The doses of SO and MBZ were chosen according to previous data^33,34^.

#### Blood and tissue sampling

At the end of the study, the mice were euthanized by decapitation, blood was collected, and serum samples were kept at −80 °C for further analysis. The livers were washed thoroughly with chilled PBS and separated into three parts. One part was stored immediately at −80 °C for later analysis of the expression of cyclin D1 mRNA by quantitative real time PCR, while the second part was stored in 10% v/v neutral buffered formalin and processed for histopathological analysis. The third part of liver tissue was homogenized in chilled buffer solution (pH 7.4), and the homogenates were centrifuged at 5000 × g for 5 minutes. The supernatants were kept at −80 °C for further biochemical testing.

#### Macroscopic and histological examination

Macroscopically visible HCC nodules greater than one mm in diameter on the surface of each liver were recorded. Parts of the collected livers were fixed in 10% v/v neutral buffered formalin for 24 h and embedded in paraffin. Histological changes were examined in 5-μm-thick sections stained with haematoxylin and eosin (H&E). HCC was graded as described by Bosman, *et al*.^35^. Images were taken using a BX51 Olympus optical microscope (Olympus Corporation, Tokyo, Japan).

#### Immunohistochemical analysis

Immunohistochemical analyses for the anti-apoptotic marker BCL-2 and the proliferation-associated marker Ki67 were performed according to methods previously described by Abdo, *et al*.^36^. The liver sections were deparaffinized, hydrated and then immersed in an antigen retrieval solution. The sections were incubated with a primary antibody against BCL-2 (Clone: BCL-w; polyclonal antibody, Zymed, San Francisco, CA, USA; dilution: 1:500) or Ki67 (rabbit polyclonal anti-Ki67 antibody (ab16502), Abcam, United Kingdom; 1:300 dilution). Then, anti-mouse or anti-rabbit IgG was used as a secondary antibody for BCL-2 or Ki67 (EnVision + System HRP, Dako), respectively. The sections were examined microscopically for specific staining, and photographs were acquired using a digital image capture system. The labelling indices for BCL-2 and Ki67 are expressed as the percentages of positive cells per 1000 counted cells in 7 to 9 high-powered fields.

#### Determination of alanine aminotransferase (ALT) activity and alpha-fetoprotein (AFP) levels

ALT serum activity was measured using a commercial kit purchased from Biodiagnostics, Cairo, Egypt. Serum levels of AFP were measured by ELISA using kits supplied by R&D Systems.

#### Determination of tumour necrosis factor alpha (TNF-α) and TGF-β1 levels

Serum levels of TNF-α and TGF-β1 were measured using ELISA kits obtained from R&D Systems (Minneapolis, MN, USA).

#### Determination of BCL-2, caspase-3 and caspase-9 levels

Caspase-3 was measured in liver tissue homogenate by ELISA using kits supplied by Uscn Life Science, Inc. (Houston, TX, USA). BCL-2 and caspase-9 levels were measured in liver tissue homogenate by ELISA using kits supplied by CUSABIO and CusAb (Wuhan, China).

#### Determination of MMP-2 and TIMP-1 levels

Following the manufacturer’s protocol, MMP-2 and TIMP-1 levels were estimated in liver tissue homogenate using ELISA kits obtained from R&D Systems.

#### Determination of VEGF, p-ERK1/2 and t-ERK1/2 levels

VEGF levels were measured in liver homogenates by ELISA using kits supplied by R&D Systems. Measurement of ERK1/2 (total and phosphorylated) levels in liver homogenates was performed by extraction using the Cell Extraction Buffer PTR provided in an ELISA kit purchased from Abcam (Cambridge, MA, USA).

#### Quantitative real-time PCR for the expression of hepatic cyclin D1

Following the manufacturer’s instructions, RNA was extracted with an RNeasy Mini kit obtained from Qiagen (Hilden, Germany) in an RNase-free environment. A Nano Drop 2000 spectrophotometer (Thermo Fisher Scientific, USA) was used for the determination of the amount and purity of RNA. One µg of RNA was reverse transcribed by Quantiscript reverse transcriptase Kit supplied by Qiagen. The polymerase chain reaction was completed using a Rotor Gene Q thermocycler (Qiagen) and SYBR Green PCR Master Mix (Qiagen). Quantitative PCR was performed in triplicate and included no template controls. The sequences of the PCR primer pairs used: cyclin D1, 5′-TGCTTGGGAAGTTGTGTTGG-3′ (forward) and 5′-AATGCCATCACGGTCCCTAC-3′ (reverse); GAPDH, 5′-TCAAGAAGGTGGTGAAGCAG-3′ (forward and 5′-AGGTGGAAGAATGGGAGTTG-3′ (reverse). The relative gene expression was assessed by the comparative cycle threshold (Ct) (2^−ΔΔCT^) method and values were normalized to the expression of GAPDH.

#### Statistical analysis

Statistical analyses were performed using GraphPad Prism software version 6 (GraphPad Software, Inc., La Jolla, CA, USA). The data are expressed as the mean ± standard deviation (SD). Differences between groups were evaluated using one-way analysis of variance (ANOVA) followed by Tukey’s multiple comparisons post-tests. With regards to survival probability, a log rank (Mantel-Cox) test was performed to assess the significance of differences between groups in Kaplan-Meier analysis. A value of P < 0.05 was considered to indicate statistical significance. The P-values were adjusted using the Hochberg’s method.

## Results

### *In vitro* antitumour activity

#### *In vitro* cytotoxicity of MBZ to HepG2 cells

As shown in Table 2, the CTC50 value for MBZ was >5 µmol/L. Therefore, doses of 0.5, 1 and 5 µmol/L were selected to further evaluate the differential effects of MBZ on the basal levels of p-MEK1/2, t-MEK1/2, p-ERK1/2 and t-ERK1/2.

**Table 2:** The cytotoxic property of MBZ on HepG2.

MBZ (µmol/L)	OD540 ± SD	% growth inhibition
0	0.588 ± 0.08	0
0.1	0.572 ± 0.07	2.72
0.5	0.512 ± 0.05	12.92
1	0.401 ± 0.04	31.8
5	0.201 ± 0.04	65.8
CTC50 (µmol/L)		>5

CTC, cytotoxic concentration; MBZ, mebendazole; OD, optical density.

#### Effect of MBZ on p-MEK1/2, t-MEK1/2, p-ERK1/2 and t-ERK1/2 in HepG2 cells

**Figure 1:**
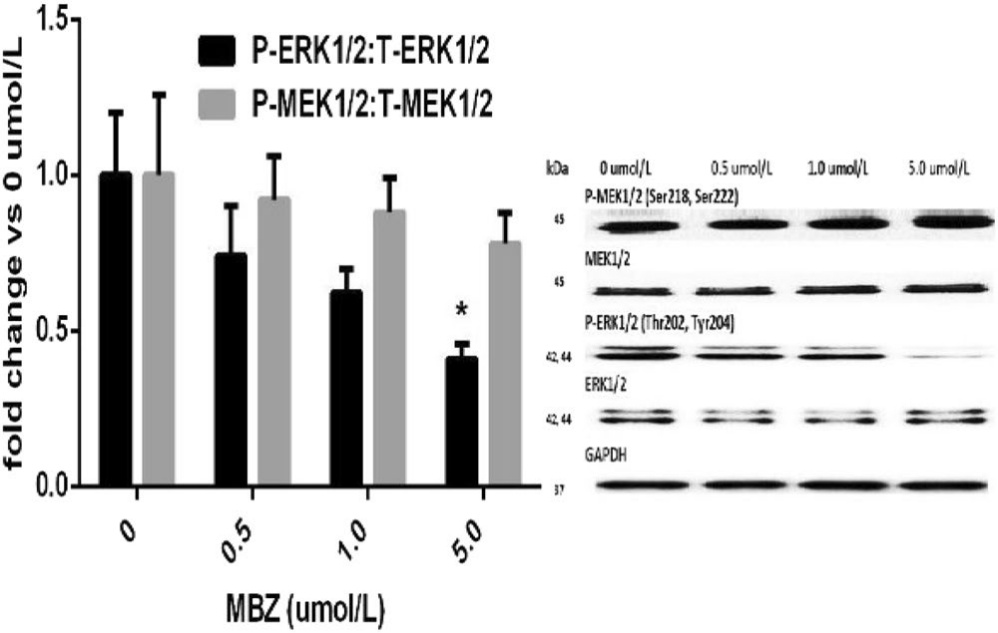
Effects of different concentrations of MBZ on p-MEK1/2:t-MEK1/2 and p-ERK1/2:t-ERK1/2 ratios in HepG2 cells (as measured by western blot analysis). The data are presented as the mean ± SD (n = 3) of the fold change in the ratio vs. that of untreated HepG2 cells. Statistical analysis was performed using ordinary one-way ANOVA followed by Tukey’s post-test. *P < 0.05 vs. untreated HepG2 cells.

As depicted in Fig. 1, cells treated with 5 µmol/L MBZ demonstrated significant reductions in the relative expression of p-ERK1/2 (as measured by western blot analysis) compared with untreated cells (P < 0.05). In addition, a trend toward decreased levels was observed in cells treated with 0.5 and 1 µmol/L MBZ. However, none of the MBZ doses significantly changed the relative expression of p-MEK1/2 (P < 0.05) indicating that MBZ targeted only the final step of the MAPK pathway.

### *In vivo* antitumour activity

#### Effect of drug treatment on DEN-induced histological changes

**Figure 2:**
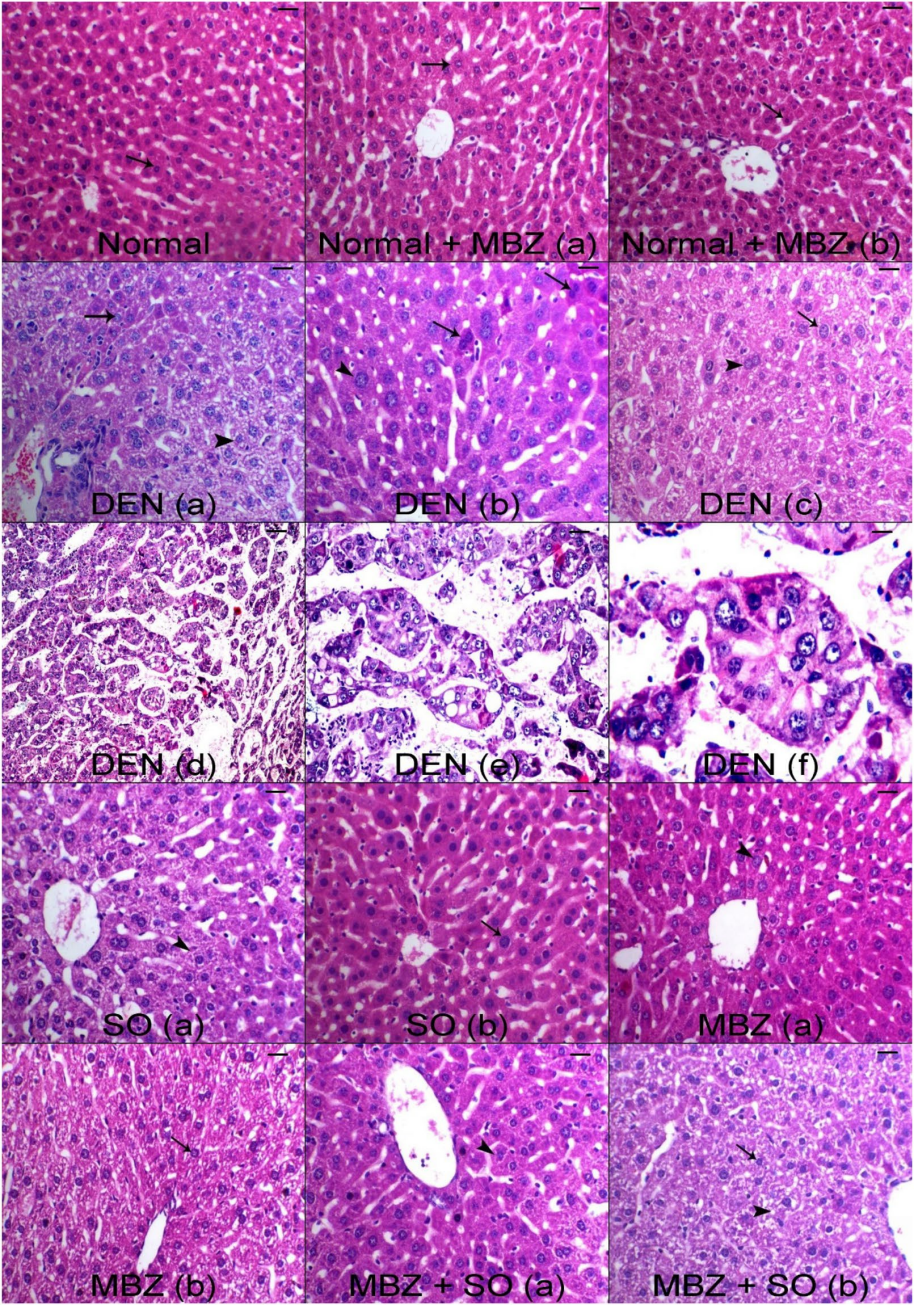
Representative light micrographs from (normal, normal + MBZ a) untreated normal and normal + MBZ control mice showing normal hepatocytes arranged in cords around the central vein (arrow) (H&E, scale bar: 100 µm); (DEN **a**) DEN-treated model mice showing basophilic preneoplastic foci (arrow) and centrilobular cytoplasmic vacuolation (arrowhead), (DEN **b**) DEN-treated model mice showing nucleomegaly (arrowhead) and increases in binuclear hepatocytes (arrows); (DEN **c**) DEN-treated model mice showing hepatocyte hypertrophy associated with nucleomegaly (arrow) and increases in binuclear hepatocytes (arrowhead) (H&E, scale bar: 100 µm); (DEN **d–f**) DEN-treated model mice showing groups of anaplastic cells replacing hepatic parenchyma forming glandular pattern of HCC in focal manner (H&E, scale bar: 100, 50, 25 µm, respectively); (SO **a,b**) SO-treated HCC mice showing centrilobular vacuolation (arrowhead) and decreases in nuclear enlargement (arrow) (H&E, scale bar: 100 µm); (MBZ **a,b**) MBZ-treated HCC mice showing decreases in both hepatic basophilic altered cells and nucleomegaly features (arrowhead) and showing mild hepatic vacuolation with normal nuclear features (arrow) (H&E, scale bar: 100 µm); (MBZ + SO **a**) MBZ + SO-treated HCC mice showing marked decreases in malignant basophilic altered cells and nucleomegaly (arrowhead); and (MBZ + SO **b**) MBZ + SO-treated HCC mice showing marked increase in hepatic vacuolation (arrowhead) and pyknotic nuclei (karyopyknosis, which is an irreversible condensation of chromatin in the nucleus of a cell undergoing necrosis or apoptosis) as well as appearance of areas of degeneration (cytoplasmic shrinkage and condensation related to cell death) (H&E, scale bar: 100 µm).

As represented in Fig. 2, tissue sections from the normal group displayed normal liver histology and a radial arrangement of hepatic parenchyma. Additionally, tissue sections from the normal + MBZ group showed normal hepatocytes arranged in cords around the central vein (normal + MBZ a, b). On the other hand, liver specimens from the DEN-treated model mice (DEN a, b, c) displayed basophilic preneoplastic foci and centrilobular cytoplasmic vacuolation as well as nucleomegaly and increases in binuclear hepatocytes associated with hepatocyte hypertrophy and (DEN d, e, f) displayed groups of anaplastic cells forming glandular pattern of HCC in focal manner all over the parenchyma with the presence of apoptotic cells and karyopyknosis (an irreversible condensation of chromatin in the nucleus of a cell undergoing necrosis or apoptosis) as well as appearance of areas of degeneration (cytoplasmic shrinkage and condensation related to cell death). Treatment of HCC mice with SO (SO a, b) decreased both centrilobular vacuolation and nuclear enlargement. The MBZ-treated HCC group (MBZ a, b) showed marked decreases in both hepatic basophilic altered cells and nucleomegaly accompanied by normal nuclear conformations and mild hepatic vacuolation, while the HCC group treated with both MBZ and SO (MBZ + SO a, b) displayed marked regression of malignant basophilic altered cells and nucleomegaly.

#### Effect of drug treatment on BCL-2 and Ki67 immunolabelling

**Figure 3:**
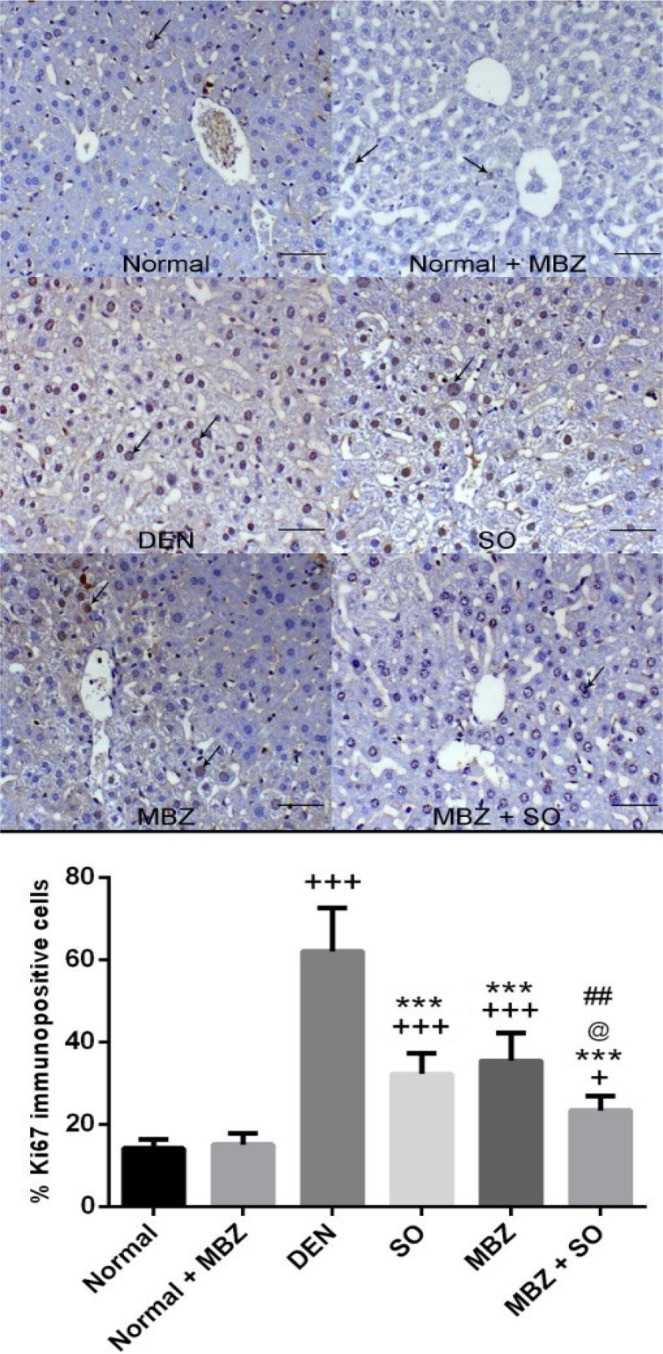
Expression of Ki67 in liver tissue from normal and normal + MBZ control mice showing few hepatocytes and nuclear expression of Ki67 (arrows), from DEN-treated model mice showing diffuse Ki67 immunostaining within hepatocytes (arrows), from SO-treated HCC mice showing decreased hepatic Ki67 immunostaining (the arrow indicates centrilobular expression), from MBZ-treated HCC mice showing decreased hepatic Ki67 expression (arrows), and from MBZ + SO-treated HCC mice showing marked decreases in hepatic Ki67 immunostaining (arrow) (Ki67 immunohistochemistry (IHC), scale bar: 200 µm). The positive cells were counted to record the expression (as the percentage of immunopositive cells). The results are expressed as the mean ± SD. ^+^P < 0.05 vs. normal, ^+++^P < 0.001 vs. normal, ***P < 0.001 vs. DEN, @ P < 0.05 vs. SO, and ^##^P < 0.01 vs. MBZ.

**Figure 4:**
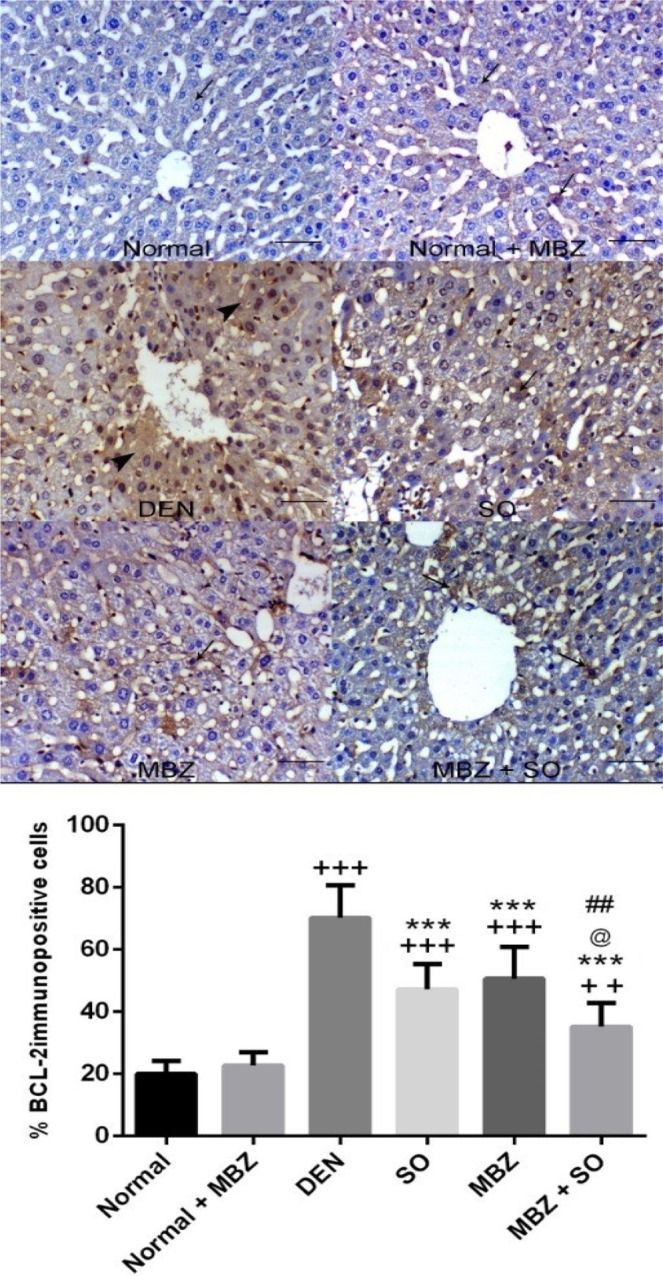
Expression of BCL-2 in liver tissue from normal and normal + MBZ control mice showing mild hepatic cytoplasmic expression of BCL-2 within hepatocytes (arrow), from DEN-treated model mice showing increases in centrilobular BCL-2 immunostaining, from SO-treated HCC mice showing decreased BCL-2 expression (arrow), from MBZ-treated HCC mice showing decreased hepatic BCL-2 expression (arrow), and from MBZ + SO-treated HCC mice showing marked decreases in the number of hepatocytes expressing BCL-2 (arrows) (Bcl-2 IHC, scale bar: 200 µm). The positive cells were counted to record the expression (as the percentage of immunopositive cells). The results are expressed as the mean ± SD. ^++^P < 0.01 vs. normal, ^+++^P < 0.001 vs. normal, ***P < 0.001 vs. DEN, @ P < 0.05 vs. SO, and ^##^P < 0.01 vs. MBZ.

As shown in Fig. 3, tissue sections from the normal group and the normal + MBZ group displayed mild cytoplasmic expression of BCL-2 within hepatocytes. However, liver specimens from the DEN-treated model mice presented centrilobular increases in the anti-apoptotic marker (BCL-2) immunostaining. Treatment of HCC mice with either SO or MBZ induced cell death as indicated by decreased expression of the anti-apoptotic marker BCL-2 (P < 0.001). However, combined treatment with SO and MBZ significantly decreased BCL-2 expression compared to monotherapy. Figure 4 illustrates the immunostaining results for the proliferation-associated marker Ki67. Tissue sections from the normal group showed nuclear expression of Ki67 antibodies within hepatocytes, while HCC mice (DEN) showed diffuse Ki67 immunostaining within hepatocytes; this immunostaining was significantly decreased in both SO- and MBZ-treated mice (P < 0.001).

#### Effect of drug treatment on serum ALT and AFP levels and tumour burden

**Figure 5:**
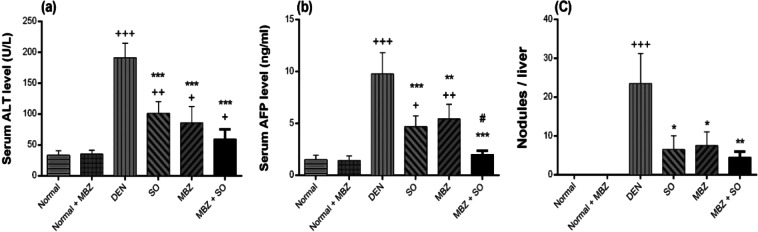
Effect of MBZ (100 mg/kg), SO (30 mg/kg) and the combination of both on the serum levels of (**a**) ALT, (**b**) AFP and (**c**) the number of nodules per liver in mice with DEN-induced HCC. The data are expressed as the mean ± SD (n = 4). Statistical analysis was performed using ordinary one-way ANOVA followed by Tukey’s post hoc test. ^+^P < 0.05 vs. normal, ^++^P < 0.01 vs. normal, ^+++^P < 0.001 vs. normal, *P < 0.05 vs. DEN, **P < 0.01 vs. DEN, ***P < 0.001 vs. DEN, and ^#^P < 0.05 vs. MBZ.

Serum levels of ALT and AFP were significantly higher (P < 0.001) in DEN-treated model mice than in normal mice. However, these values were considerably lower in mice treated with SO (P < 0.001), MBZ (P < 0.001 for ALT and P < 0.01 for AFP) or the combination of both (P < 0.001) than in DEN-only mice (Fig. 5a,b). Similarly, SO, MBZ and the combination of both significantly decreased the DEN-induced increases in the number of hepatic nodules (Fig. 5c).

#### Effect of drug treatment on serum TNF-α and TGF-β1 levels

**Figure 6:**
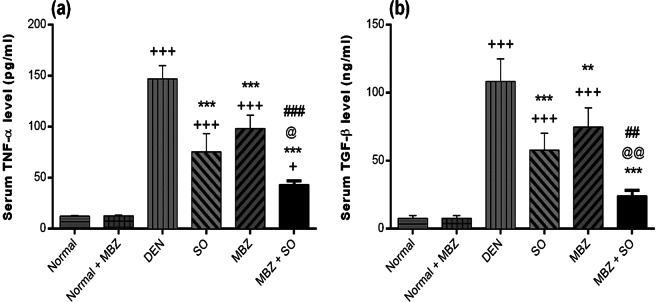
Effect of MBZ (100 mg/kg), SO (30 mg/kg) and the combination of both on the serum levels of (**a**) TNF-α and (**b**) TGF-β in mice with DEN-induced HCC. The data are expressed as the mean ± SD (n = 4). Statistical analysis was performed using ordinary one-way ANOVA followed by Tukey’s post-test. ^+^P < 0.05 vs. normal, ^+++^P < 0.001 vs. normal, **P < 0.01 vs. DEN, ***P < 0.001 vs. DEN, @ P < 0.05 vs. SO, @@ P < 0.01 vs. SO, ^##^P < 0.01 vs. MBZ, and ^###^P < 0.001 vs. MBZ.

Serum levels of TNF-α and TGF-β1 were significantly higher (P < 0.001) in DEN-treated model mice than in normal mice. However, these values were considerably lower in mice treated with SO (P < 0.001), MBZ (P < 0.001 for TNF-α, P < 0.01 for TGF-β1) or the combination of both (P < 0.001) than in DEN-only mice (Fig. 6). Furthermore, MBZ + SO treatment significantly reduced the levels of these proteins compared with either SO or MBZ monotherapy.

#### Effect of drug treatment on hepatic BCL-2, caspase-3 and caspase-9 levels

**Figure 7:**
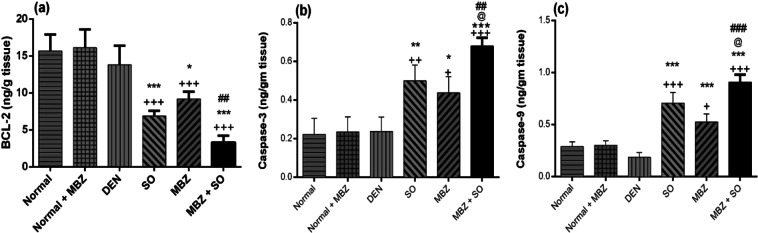
Effect of MBZ (100 mg/kg), SO (30 mg/kg) and the combination of both on the hepatic levels of (**a**) BCL-2, (**b**) caspase-3 and (**c**) caspase-9 in mice with DEN-induced HCC. The data are expressed as the mean ± SD (n = 4). Statistical analysis was performed using ordinary one-way ANOVA followed by Tukey’s post-test. ^+^P < 0.05 vs. normal, ^++^P < 0.01 vs. normal, ^+++^P < 0.001 vs. normal, *P < 0.05 vs. DEN, **P < 0.01 vs. DEN, ***P < 0.001 vs. DEN, @ P < 0.05 vs. SO, ^##^P < 0.01 vs. MBZ, and ^###^P < 0.001 vs. MBZ.

As depicted in Fig. 7, DEN-treated model mice did not exhibit significant differences in the levels of BCL-2, caspase-3 and caspase-9 compared to normal mice. However, the levels of the anti-apoptotic marker BCL-2 were significantly lower in the drug-treated HCC groups than in the normal or DEN-treated model groups (P < 0.001). The induced-cell death was suggested by the significant increase in caspase-3 and caspase-9 levels in drug-treated HCC mice compared to normal or DEN-treated model mice. Moreover, the MBZ + SO group demonstrated significantly lower BCL-2 levels than the MBZ monotherapy group (P < 0.01) and significantly higher caspase-3 and caspase-9 levels than either the MBZ or SO monotherapy group.

#### Effect of drug treatment on hepatic cyclin D1 mRNA expression

**Figure 8:**
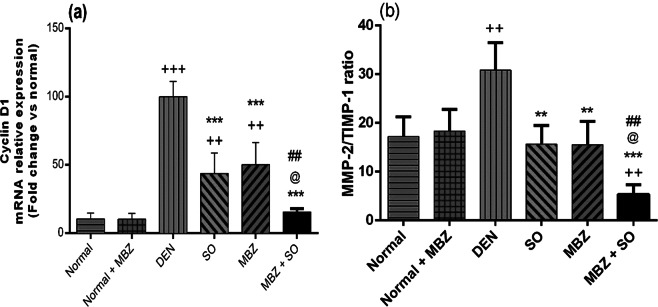
Effect of MBZ (100 mg/kg), SO (30 mg/kg) and the combination of both on (**a**) hepatic cyclin D1 mRNA relative expression (data expressed as the fold change from the normal control value after normalization by GAPDH ± SD, n = 4) and (**b**) the hepatic MMP-2:TIMP-1 ratio (data expressed as the mean of the ratio ± SD, n = 4) in mice with DEN-induced HCC. Statistical analysis was performed using ordinary one-way ANOVA followed by Tukey’s post-test. ^++^P < 0.01 vs. normal, ^+++^P < 0.001 vs. normal, **P < 0.01 vs. DEN, ***P < 0.001 vs. DEN, @ P < 0.05 vs. SO, and ^##^P < 0.01 vs. MBZ.

Figure 8a shows that cyclin D1 mRNA expression was significantly higher in the DEN-treated model mice than in the normal mice (P < 0.001). However, the drug-treated HCC groups exhibited significantly lower expression than the DEN-treated model group (P < 0.001). Furthermore, MBZ + SO-treated mice demonstrated significantly lower cyclin D1 gene expression than mice receiving either MBZ (P < 0.01) or SO (P < 0.05) monotherapy.

#### Effect of drug treatment on the hepatic ratio of MMP-2 and TIMP-1

Figure 8b shows that the MMP-2:TIMP-1 ratio was significantly higher in the DEN-treated model mice than in the normal mice (P < 0.01). However, the drug-treated HCC groups exhibited significantly lower ratios than the DEN-treated model group (P < 0.01 for MBZ or SO, P < 0.001 for MBZ + SO). Moreover, MBZ + SO-treated mice demonstrated significantly lower ratios than mice receiving either MBZ (P < 0.01) or SO (P < 0.05) monotherapy. Table 3 further shows how cell cycle progression (as indicated by hepatic cyclin D1 gene expression) and tumour cell proliferation (as indexed by Ki67 immunolabelling) were strongly and significantly correlated with ECM production and tumour cell dissemination tendency (as assessed by the MMP-2:TIMP-1 ratio) (P < 0.001). Hepatic cyclin D1 gene expression was negatively correlated with caspase-9 level (P < 0.05).

**Table 3:** Correlation between tumor cellular proliferations, tumor angiogenesis and cell cycle progression marker (Ki-67 immunolabeling, VEGF level and Cyclin D gene expression, respectively), tumor cell metastasis tendency (MMP-2/TIMP-1 ratio) and caspase-9.

Correlated parameters		R	P
Ki67 immunolabeling	MMP-2/TIMP-1 ratio	0.706	0.00
VEGF level	MMP-2/TIMP-1 ratio	0.745	0.00
	caspase-9	−0.484	0.031
Hepatic cyclin D1 gene expression	MMP-2/TIMP-1 ratio	0.786	0.00
	caspase-9	−0.498	0.02

#### Effect of drug treatment on hepatic VEGF, p-ERK1/2 and t-ERK1/2

**Figure 9:**
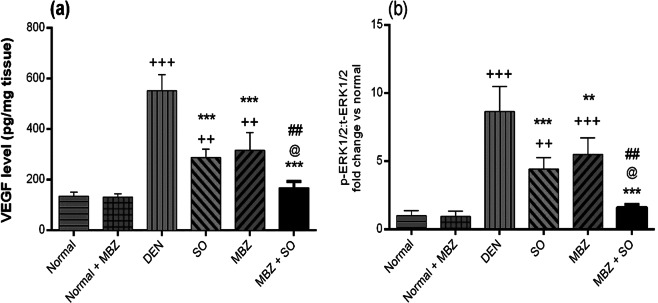
Effect of MBZ (100 mg/kg), SO (30 mg/kg) and the combination of both on the hepatic levels of (**a)** VEGF (data expressed as the mean ± SD, n = 4) and (**b**) p-ERK1/2:t-ERK1/2 (measured by ELISA; values expressed as the mean ± SD of the ratio normalized with respect to the normal control ratio, n = 4) in mice with DEN-induced HCC. Statistical analysis was performed using ordinary one-way ANOVA followed by Tukey’s post-test. ^++^P < 0.01 vs. normal, ^+++^P < 0.001 vs. normal, **P < 0.01 vs. DEN, ***P < 0.001 vs. DEN, @ P < 0.05 vs. SO, and ^##^P < 0.01 vs. MBZ.

Figure 9a shows that VEGF levels were significantly higher in the DEN-treated model mice than in the normal mice (P < 0.001). However, the drug-treated HCC groups exhibited significantly lower VEGF levels than the DEN-treated model group (P < 0.001). MBZ + SO treatment had the most significant effect. Additionally, a correlation study revealed a strong positive correlation between tumour angiogenesis (as demonstrated by VEGF levels) and tumour cell metastasis tendency (as demonstrated by the MMP-2:TIMP-1 ratio) (P < 0.001). However, the level of caspase-9 was negatively correlated with VEGF levels (P < 0.05) (Table 3). Furthermore, HCC model mice showed significantly higher p-ERK1/2:t-ERK1/2 ratios than normal mice (P < 0.001); however, treatment with SO, MBZ or a combination of both significantly decreased the ratio (P < 0.001 for SO or MBZ + SO, P < 0.01 for MBZ). Moreover, the combination of the 2 treatments exerted a more obvious effect than either SO or MBZ monotherapy (P < 0.05 and P < 0.01, respectively) (Fig. 9b).

#### Effect on survival probability

**Figure 10:**
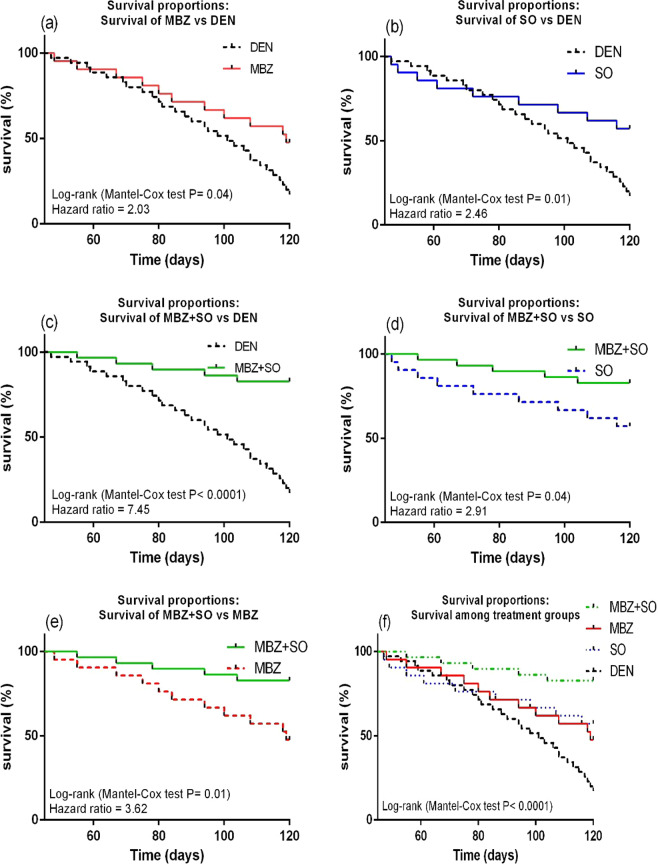
Kaplan-Meier survival plots of (**a**), MBZ vs. DEN; (**b**), SO vs. DEN; (**c**), MBZ + SO vs. DEN; (**d**), MBZ + SO vs. SO; (**e**), MBZ + SO vs. MBZ and (**f**), survival proportions between treatment groups. Statistical analysis was done using the log rank test (Mantel-cox method). P values < 0.05 were considered significant.

The Kaplan-Meier survival curves depicted in Fig. 10 reveal that DEN-treated model mice had higher mortality rates than MBZ-treated HCC mice (log-rank test P-adj = 0.04, hazard ratio = 2.03) (Fig. 10a), SO-treated HCC mice (log-rank test P-adj = 0.03, hazard ratio = 2.46) (Fig. 10b), and MBZ + SO-treated HCC mice (log-rank test P-adj = 0.0005, hazard ratio = 7.45) (Fig. 10c), indicating that the drug-treated HCC mice exhibited higher survival curves and survival % than the DEN-treated model mice. In addition, the MBZ + SO group demonstrated the highest survival probability among the treatment groups, as illustrated in Fig. 10d (MBZ + SO vs. SO: log-rank test P-adj = 0.04, hazard ratio = 2.91) and Fig. 10e (MBZ + SO vs. MBZ: log-rank test P-adj = 0.03, hazard ratio = 3.62)

## Discussion

There has been conflicting evidence concerning the effect of MBZ on tumour neo-vascularization^34^. In addition, it is probable that the antitumour activity of MBZ is mediated by molecular targets yet to be clarified. Our objective was to generate novel, multi-targeted treatment options with minimal risk of toxicity. One prospective treatment is the use of an oral microtubule-disrupting agent with apoptotic and anti-angiogenic activity that could target the final step of the Ras/Raf/MEK/ERK signalling pathway. Such a treatment could minimize resistance to SO. Hence, combination therapy with MBZ with SO may be a promising avenue for exploration.

In the present study, the fundamental findings included the remarkable efficacy of MBZ in inhibiting ERK phosphorylation (*in vitro* and *in vivo*) and subsequently inhibiting target genes such as VEGF and TGFβ-1. However, dual therapy with SO elicited more noticeable results than MBZ monotherapy. It has been documented that SO does not increase survival by more than 3–4 months^7^. Interestingly, our results demonstrated that combined treatment with MBZ and SO markedly increased mouse survival compared with MBZ or SO monotherapy.

It is generally accepted that the RAS/RAF/MEK/ERK signalling pathway plays a substantial role in the incidence and development of HCC^10^. Generally, there is a strong relationship between this pathway and the overexpression of many growth factors, including VEGF. VEGF and RTK interaction results in activation of the RAS/RAF/MEK/ERK pathway^37^. In addition, tumour growth and invasion are dependent on deregulated angiogenesis. Consequently, many therapeutic agents targeting VEGFR, such as the multi-kinase inhibitor SO, are currently being developed for HCC treatment^8,38^. Our study revealed that MBZ possesses anti-angiogenic activity and augments the anti-angiogenic activity of SO, an effect that seems to be due to inhibition of the RAS/RAF/MEK/ERK pathway at the final step (ERK1/2 phosphorylation). This result was also confirmed by our *in vitro* finding that MBZ treatment did not significantly change the relative expression of p-MEK1/2 compared to no treatment.

TGF-β induces the RAS/RAF/MEK/ERK signalling pathway, and inactivation of p38 MAPK prevents the TGF-β-induced epithelial-mesenchymal transition that accelerates carcinoma cell invasion and distribution^39^. Additionally, elevated levels of TGF-β are associated with increased angiogenesis due to induction of TGF-β-dependent VEGF expression^40^. Consistent with our results, a study by Serova, *et al*.^41^ suggested that TGF-β inhibition was effective *in vitro* and *ex vivo* in an advanced HCC model. On the other hand, it has been validated that an imbalance between MMP-2, which regulates ECM degradation, and TIMP-1, its tissue inhibitor, plays an important role in tumour invasion and cancer metastasis^42^. A previous study demonstrated that MMP-2 is activated by the RAS/ERK pathway^43,44^. Our study found that SO and MBZ, in addition to exhibiting anti-angiogenic activity (as shown by the significant reductions in VEGF levels) markedly reduced the MMP-2:TIMP-1 ratio. Combination therapy elicited a more noticeable effect than either SO or MBZ monotherapy. This finding suggests that SO and MBZ treatment can suppress tumour metastasis and invasion by inhibiting the ERK/MMP-2 pathway. This ability was further confirmed by the strong positive correlation between tumour angiogenesis, as demonstrated by VEGF levels, and ECM production and tumour cell invasion tendency, as demonstrated by the MMP-2:TIMP-1 ratio.

In the same context, the RAS/RAF/MEK/ERK cascade plays an important role in apoptosis by phosphorylating various apoptosis-regulating factors^16^. Our study revealed that treatment with MBZ alone or combined with SO induced cell death in HCC liver tissue, as evidenced by increased hepatic caspase-3 and caspase-9 levels and decreased hepatic BCL-2 levels. In addition, the visual inspection of liver specimens from different treatment groups confirmed the appearance of hepatic vacuolation and pyknotic nuclei in cells that may undergo apoptosis as well as appearance of areas displaying cytoplasmic shrinkage and condensation that might be related to cell death. Many studies have attributed the antitumour activity of MBZ to its role in apoptosis induction through BCL-2 phosphorylation, which prevents the interaction of BCL-2 with the pro-apoptotic BCL-2-associated x protein (Bax), thereby promoting apoptosis^26,45^. Additionally, many studies have suggested that the mechanism of action of MBZ as an anti-tubulin drug in cancer cells is mediated by its effect on caspase-3 and caspase-9 in different tumour types, such as melanoma and breast cancer^46,47^. It is noteworthy that increased apoptotic activity could be linked to the increased levels of total caspase-3 and -9^48–53^. Notably, the cytotoxic effect of SO is crucial for its antitumour activity. Apoptosis is the major form of this cytotoxicity and is required for tumour regression and continued clinical improvement^54^. Consistently, we identified a negative correlation between tumour angiogenesis, as corroborated by VEGF levels, and cell death as verified by caspase-9 levels.

Overexpression of cyclin D1, a key gene in cell cycle control, is generally associated with many types of tumours, including HCC^55^. Furthermore, cyclin D1 is capable of inducing instability and DNA amplification, resulting in the alteration and transformation of cells^56^. In our study, SO acted as a cell cycle inhibitor by effectively downregulating cyclin D1 gene expression. This result is in accordance with those of previous studies that have attributed the antitumour and proliferation-inhibiting effects of SO to SO-mediated cyclin D1 inhibition^31,57,58^. Our results revealed that MBZ treatment also significantly downregulated cyclin D1 gene expression and inhibited cellular proliferation in HCC mice. In combination therapy, MBZ strongly potentiated the effects of SO by inhibiting cellular proliferation through downregulation of cyclin D1 gene expression. The observed decrease in cyclin D1 expression may have been due to inhibition of the RAS/RAF/MEK/ERK pathway, as cyclin D1 is transcribed and activated through ERK phosphorylation^59,60^. Consistently, there was a significant negative correlation between hepatic cyclin D1 gene expression and cell death and a significant positive correlation between cyclin D1 expression and cancer metastatic capability (as assessed by the MMP-2:TIMP-1 ratio). These findings are consistent with a recent study reported that MBZ exhibited anticancer efficacy against acute myeloid leukaemia through mitotic arrest induction^27^.

Ki67 is a nuclear antigen expressed in proliferating cells that is used as a tumour proliferation-associated marker in HCC and that affects disease progression and prognosis^61,62^. Our results showed that tumour cell proliferation, as clarified by Ki67 immunolabelling, was significantly lower in HCC mice receiving SO, MBZ or the combination of both than in DEN-only mice. These results were further confirmed by the strong positive correlation between the number of Ki67-positive cells and the HCC metastatic ability indicated by the MMP-2:TIMP-1 ratio.

**Figure 11:**
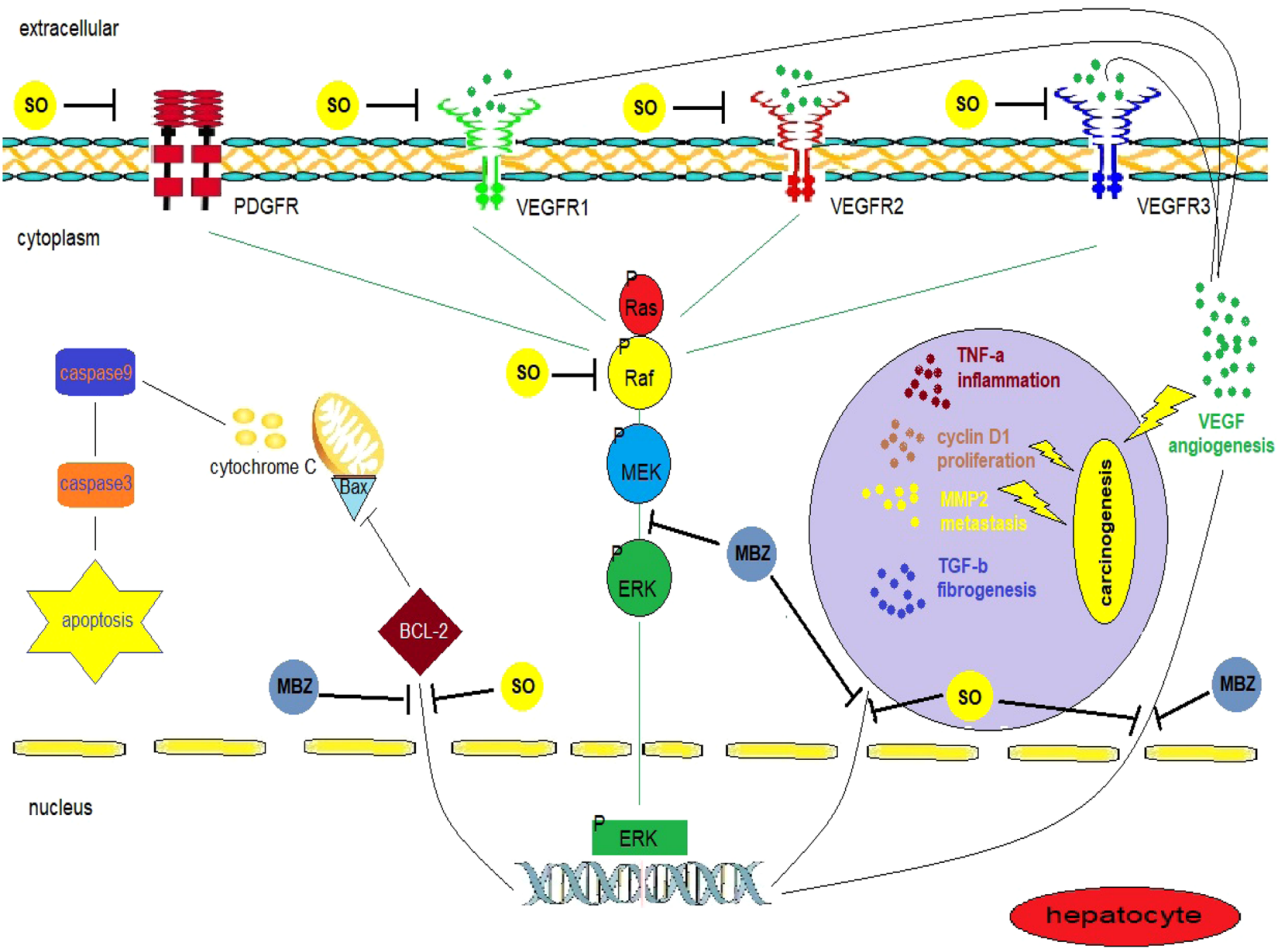
The proposed mechanism of action of mebendazole.

In the present study, restoration of liver function after MBZ treatment was indicated by a significant decrease in ALT levels. In addition, a decreased number of tumour nodules and significantly reduced levels of AFP reflected lower tumour production rates. TNF-α is frequently associated with inflammatory conditions^63–67^. In our study, the observed reductions in the levels of the inflammatory cytokine TNF-α, which were elevated in response to inflammatory cell infiltration and liver damage in HCC mice, confirmed the therapeutic effects of SO and MBZ. These results are consistent with previous reports^68,69^. Furthermore, our histopathological findings yielded evidence supporting the results of the biochemical and molecular analyses. These beneficial effects of MBZ were due to its ability to inhibit phosphorylation of ERK1/2 and its downstream target genes (Fig. 11). In this context, we have provided the first evidence that MBZ inhibits phosphorylation-induced activation of ERK1/2 *in vitro* and *in vivo*.

In our model, we treated mice with MBZ (100 mg/kg/day p.o.), using dose conversion factors, the human equivalent dose is approximately 500 mg/day (assuming a 70 kg human). In a Phase I open label study, at John Hopkins Hospital, high-grade glioma patients recruited to the trial are treated on a 28 day cycle of 500 mg MBZ tablets three times a day. Another clinical trial is at Cohen Children’s Medical Centre of New York in paediatric patients with low-grade gliomas. The MBZ dose was 100 mg twice a day over the 70 weeks of treatment^34^. In addition, clinical reports have highlighted the clinical benefit of mebendazole in patients with metastatic adrenocortical cancer, achieving observed regression in metastatic lesions (Dobrosotskaya *et al*., 2011). Another report of metastatic colon treated with mebendazole with complete regression of metastatic lesions in lungs and lymph nodes (Nygren and Larsson, 2014). Noteworthy, mebendazole in these clinical reports was well tolerated by the patient without any side effects at the standard antihelminthic dose of 100 mg twice daily to be continued for 19 months in the former case and for six weeks in the latter case. These data indicates that administration of MBZ is clinically feasible and because there is no drug interactions have been reported about MBZ and SO, MBZ is a promising adjunct candidate to SO.

Taken together, our results suggest that MBZ is a promising drug for HCC treatment with no major side effects if used in combination with SO, that potentiates the anti-metastatic effects of SO, increases survival probability and minimizes resistance. The mechanism of these beneficial effects includes inhibition of ERK phosphorylation, a final step in the RAS/RAF/MEK/ERK pathway; inhibition of cellular proliferation and cell death stimulation. Thus, this low-cost agent is a strong candidate for drug repurposing as an oncological treatment both in monotherapy and in combination therapy with currently used agents such as SO.
