# Author Correction: Mebendazole augments sensitivity to sorafenib by targeting MAPK and BCL-2 signalling in n-nitrosodiethylamine-induced murine hepatocellular carcinoma

**DOI:** 10.1038/s41598-022-17580-7

**Published:** 2022-08-10

**Authors:** Nancy S. Younis, Amal M. H. Ghanim, Sameh Saber

**Affiliations:** 1 Department of Pharmaceutical Sciences, College of Clinical Pharmacy, King Faisal University, Al-Ahsa, Kingdom of Saudi Arabia; 2 Department of Pharmacology, Zagazig University, Zagazig, Egypt; 3 Department of Biochemistry, Faculty of Pharmacy, Delta University for Science and Technology, Gamasa, Egypt; 4 Department of Pharmacology, Faculty of Pharmacy, Delta University for Science and Technology, Gamasa, Egypt

Correction to: *Scientific Reports* 10.1038/s41598-019-55666-x, published online 13 December 2019

The original version of this Article contained errors.

The P-values for the comparisons shown in Figure 10 and reported in the text were not controlled for alpha error. We used Hochberg’s method (the modified Bonferroni method) for P-value correction; the main results remain the same after this correction, but the Article now reports adjusted P-values. The Article changed as follows. The original Figure 10 and accompanying legend appear below.

**Figure 10:**
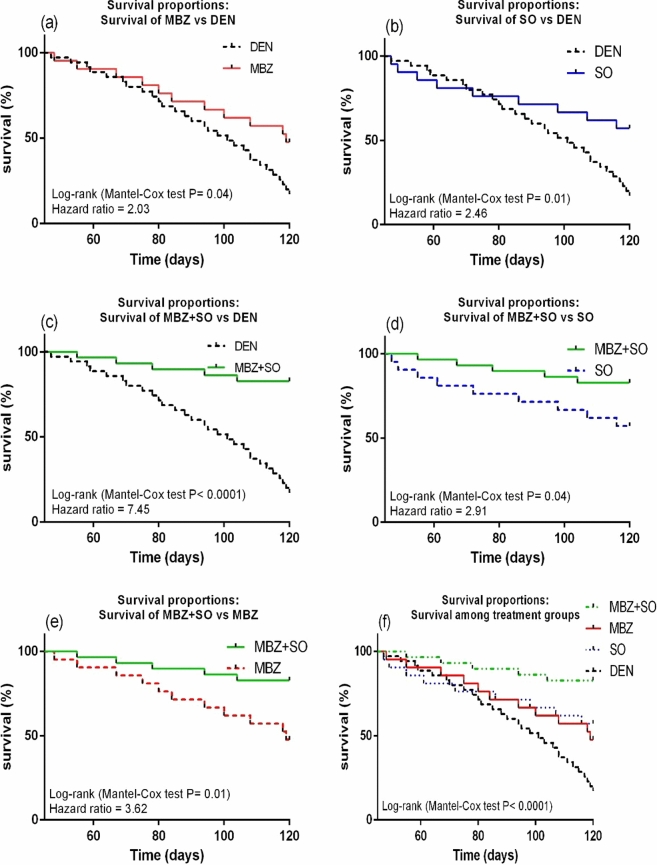
Kaplan–Meier survival plots of (**a**), MBZ vs. DEN; (**b**), SO vs. DEN; (**c**), MBZ + SO vs. DEN; (**d**), MBZ + SO vs. SO; (**e**), MBZ + SO vs. MBZ and (**f**), survival proportions between treatment groups. Statistical analysis was done using the log rank test (Mantel-cox method). P values < 0.05 were considered significant.

In the Methods, under the subheading ‘Statistical analysis’,

“With regards to survival probability, a log rank (Mantel–Cox) test was performed to assess the significance of differences between groups in Kaplan–Meier analysis. A value of P < 0.05 was considered to indicate statistical significance.”

now reads:

“With regards to survival probability, a log rank (Mantel–Cox) test was performed to assess the significance of differences between groups in Kaplan–Meier analysis. A value of P < 0.05 was considered to indicate statistical significance. The P-values were adjusted using the Hochberg's method.”

In the Results, under the subheading ‘Effect on survival probability’,

“The Kaplan–Meier survival curves depicted in Fig. 10 reveal that DEN-treated model mice had higher mortality rates than MBZ-treated HCC mice (log-rank test P = 0.04, hazard ratio = 2.03) (Fig. 10b), SO-treated HCC mice (log-rank test P = 0.01, hazard ratio = 2.46) (Fig. 10b), and MBZ + SO-treated HCC mice (log-rank test P < 0.0001, hazard ratio = 7.45) (Fig. 10c), indicating that the drug-treated HCC mice exhibited higher survival curves and survival % than the DEN-treated model mice. In addition, the MBZ + SO group demonstrated the highest survival probability among the treatment groups, as illustrated in Fig. 10f (MBZ + SO vs. SO: log-rank test P = 0.04, hazard ratio = 2.91) and Fig. 10e (MBZ + SO vs. MBZ: log-rank test P = 0.01, hazard ratio = 3.62).”

now reads:

“The Kaplan–Meier survival curves depicted in Fig. 10 reveal that DEN-treated model mice had higher mortality rates than MBZ-treated HCC mice (log-rank test P-adj = 0.04, hazard ratio = 2.03) (Fig. 10a), SO-treated HCC mice (log-rank test P-adj = 0.03, hazard ratio = 2.46) (Fig. 10b), and MBZ + SO-treated HCC mice (log-rank test P-adj = 0.0005, hazard ratio = 7.45) (Fig. 10c), indicating that the drug-treated HCC mice exhibited higher survival curves and survival % than the DEN-treated model mice. In addition, the MBZ + SO group demonstrated the highest survival probability among the treatment groups, as illustrated in Fig. 10d (MBZ + SO vs. SO: log-rank test P-adj = 0.04, hazard ratio = 2.91) and Fig. 10e (MBZ + SO vs. MBZ: log-rank test P-adj = 0.03, hazard ratio = 3.62).”

Additionally, Figure 10 was revised to include adjusted P-values.

The original Article has been corrected.

